# An AGT-based *protein-tag* system for the labelling and surface immobilization of enzymes on *E. coli* outer membrane

**DOI:** 10.1080/14756366.2018.1559161

**Published:** 2019-02-06

**Authors:** Rosa Merlo, Sonia Del Prete, Anna Valenti, Rosanna Mattossovich, Vincenzo Carginale, Claudiu T. Supuran, Clemente Capasso, Giuseppe Perugino

**Affiliations:** a Department of Biology Agriculture and Food Sciences, Institute of Bioscience and BioResources – National Research Council of Italy, Naples, Italy;; b Neurofarba Department, University of Florence, Polo Scientifico, Sesto Fiorentino Firenze, Italy

**Keywords:** Carbonic anhydrase, *β*-glycoside hydrolase, thermostable *protein-tag*, ice nucleation protein, enzyme immobilisation

## Abstract

The use of natural systems, such as outer membrane protein A (OmpA), phosphoporin E (PhoE), ice nucleation protein (INP), etc., has been proved very useful for the surface exposure of proteins on the outer membrane of Gram-negative bacteria. These strategies have the clear advantage of unifying in a one-step the production, the purification and the *in vivo* immobilisation of proteins/biocatalysts onto a specific biological support. Here, we introduce the novel *Anchoring-and-Self-Labelling-protein-tag* (ASL*^tag^)*, which allows the *in vivo* immobilisation of enzymes on *E. coli* surface and the labelling of the neosynthesised proteins with the engineered alkylguanine-DNA-alkyl-transferase (H^5^) from *Sulfolobus solfataricus*. Our results demonstrated that this *tag* enhanced the overexpression of thermostable enzymes, such as the carbonic anhydrase (*Ss*pCA) from *Sulfurihydrogenibium yellowstonense* and the *β*-glycoside hydrolase (*Ss*βGly) from *S. solfataricus,* without affecting their folding and catalytic activity, proposing a new tool for the improvement in the utilisation of biocatalysts of biotechnological interest.

## 1. Introduction

**Figure 1.**
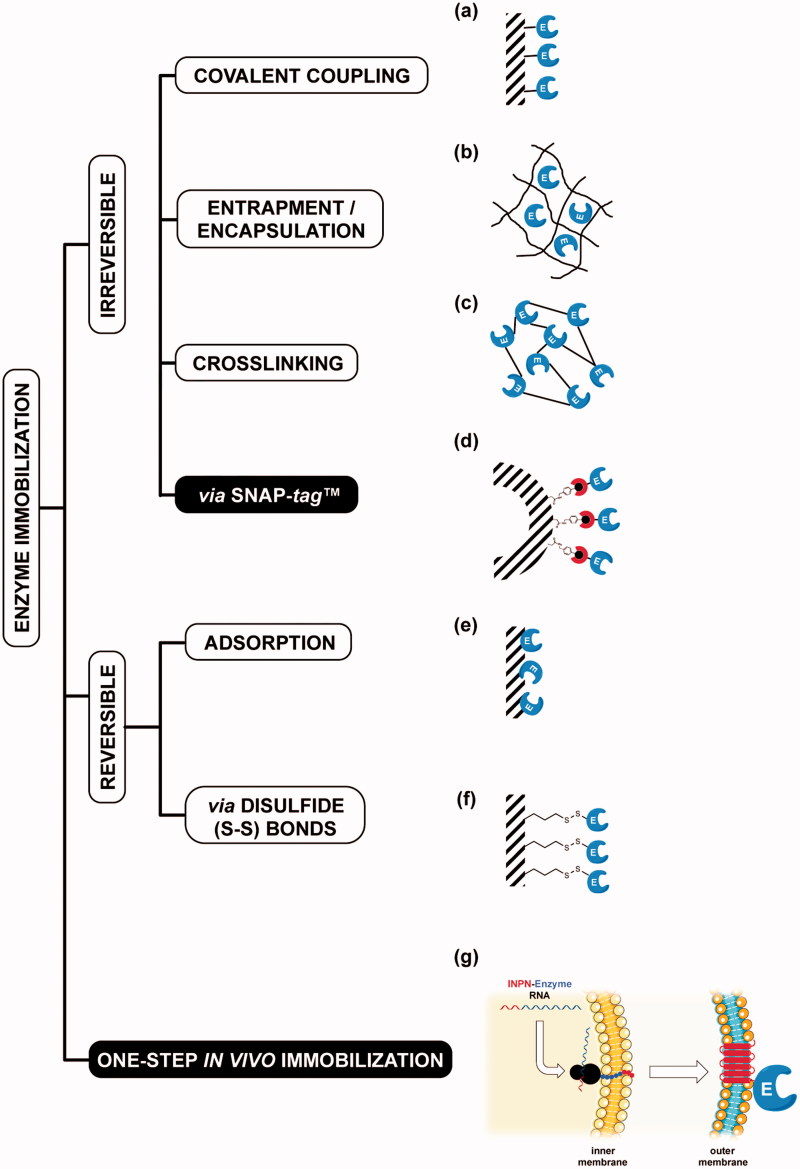
Examples of enzyme immobilisation methods. Among the traditional methods for the irreversible immobilisation (a–c, e–f) of an enzyme (E, *in blue*), the recent introduction of the SNAP-*tag*™ technology (d, *red semicircle*) allowed an indirect immobilisation of protein of interest. The *one step in vivo immobilisation* of an enzyme (g) is possible when it is expressed as fusion protein with the N-terminal domain of the ice nucleation protein (INPN, *in red*). Because of the presence of a peptide leader upstream the coding sequence, the nascent polypeptide is translocated to the cell OM by the anchoring transmembrane INPN domain, leading to the immobilisation and exposition of the biocatalyst outside the cell.

**Figure 2.**
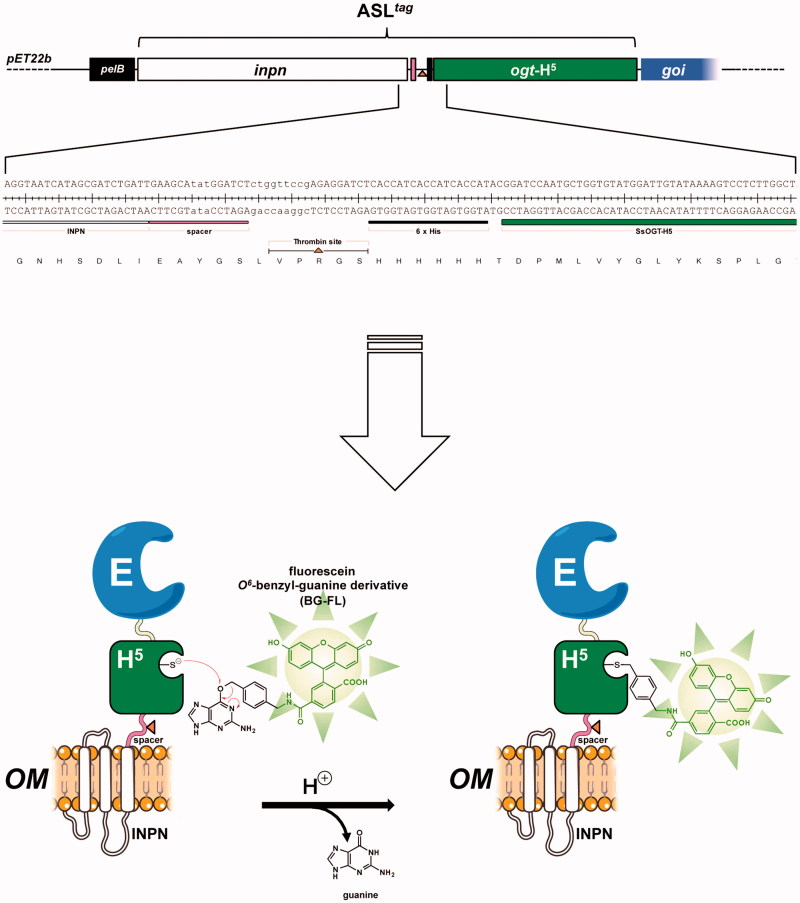
The ASL*^tag^* protein. The ASL*^tag^* gene is composed by the *inpn* ORF (*in white*) in frame fused to the *ogt*H^5^ gene (*in green*) in the pET22b expression vector. This *tag* can be further fused to a gene of interest (*goi*, *in blue*), for a *one step procedure* of the expression and immobilisation of an enzyme (E). The presence of the H^5^ moiety allows the quantitative estimation of the yield of E by the irreversible alkyl-transferase assay using a fluorescent *O^6^*-benzyl-guanine derivative (BG-FL). Between *inpn* and *ogt*H^5^, a spacer (*in pink*), a thrombin cleavage site (shown as an *orange triangle*) and a 6 × His-tag (*in black*) were inserted, for the easy separation and purification of any H^5^-E fusion protein.

The term *immobilised enzymes* refers to ‘enzymes physically confined or localised in a certain defined region of space with retention of their catalytic activities, and which can be used repeatedly and continuously’^1^. The immobilisation of enzymes on solid supports is historically very important for overcoming their general instability in harsh operational conditions and their low shelf-life, as well as the need for their recycling more times^2^. Furthermore, the physical separation of the biocatalyst from the reaction mixture avoids the protein contamination of the products. Although a reduction in reaction rates sometimes occurs, because the enzyme cannot mix freely with the substrate or a particular conformational change is needed for the biocatalyst efficiency, there are many examples of increased activity and stability of immobilised enzymes^3^. Many chemical or physical methods for the enzyme immobilisation are currently available, from the physical adsorption to the covalent coupling on supports (Figure 1(a–c,e–f))^3–6^. Recently, the use of *protein-tags* based on an engineered version of the human *O^6^*-alkyl-guanine-DNA-alkyl-transferases (hAGT) is an effective alternative for the covalent immobilisation of proteins and enzymes (Figure 1(d))^7–9^. AGTs (or OGTs, MGMTs; E.C.: 2.1.1.63) are DNA repair enzymes, which *irreversibly* transfer the alkyl group from the damaged DNA containing *O^6^*-alkyl-guanines to their cysteine residue in the active site^10–13^. In 2003, Johnsson and his group demonstrated that most enzymes of this class display relatively low substrate specificity, making them reactive also with free *O^6^*-benzyl-guanines (*O^6^*-BG) nucleobases^14^. This led to the development of the so-called SNAP-*tag*™ technology, which uses derivatives of *O^6^*-BG potentially conjugated with an unlimited number of chemical groups^15–18^. This system allows the immobilisation on *O^6^*-BG-derivatised surface of the protein expressed in fusion with the SNAP-*tag* ^18^ (Figure 1(d)). However, all these approaches mainly depend on the high costs due to the isolation and purification of the biocatalysts. This limitation can be easily overcome by the heterologous expression of enzymes and their *in vivo* direct immobilisation on the surface of bacterial hosts, by the utilisation of transmembrane protein domains, as the ice nucleation protein (INP) of the Gram-negative bacterium *Pseudomonas syringae* (Figure 1(g))^19^ ^,^ ^20^. This protein is composed of an N-terminal domain (N, 175 residues) structurally separated from a C-terminal domain (C, 49 residues) by a repetitive central domain^21^. Both domains play a role in the anchoring of proteins to the outer membrane^21^. The use of INP as *anchoring carrier* is considered of great interest in biotechnological applications, ranging from the development of bacterial cell surface-display systems for vaccine delivery to the fabrication of whole-cell biocatalysts and biosensors^22–24^. The N-terminal domain of INP (INPN) was recently and successfully used for the one-step procedure immobilisation (Figure 1(g))^22–29^. Moreover, Capasso *et al.* demonstrated that the amount of a thermostable carbonic anhydrase^30–33^ fused to the INPN domain and expressed on the bacterial cell surface had a hydratase activity similar to that of the enzyme covalently immobilised onto magnetic nanoparticles^30^ ^,^ ^34^. Here, we introduce a novel *protein-tag* system, (hereinafter *Anchoring-and-Self-Labelling-protein-tag or* ASL*^tag^*), which simultaneously allows the *in vivo* immobilisation of the enzyme of interest on the *E. coli* surface and its quantitative determination (Figure 2). The ASL*^tag^* system is formed by the INPN domain fused to an engineered and thermostable variant of the alkylguanine-DNA-alkyl-transferase (H^5^) from the hyperthermophilic archaeon *Sulfolobus solfataricus* ^35^ ^,^ ^36^. This enzyme was extensively characterised, suggesting its biotechnological role as thermostable alternative to the commercial SNAP-*tag*™ and its utilisation as *protein-tag* for heterologous expression of proteins of interest in *E. coli* and, for the first time, in thermophilic organisms as *Thermus thermophilus* and *Sulfolobus islandicus* ^35–37^. Thus, using the substrate of H^5^, a fluorescein derivative of the *O^6^*-BG (Figure 2), we successfully estimated the expression of the ASL*^tag^* in *E. coli* cells, by *in vitro gel-imaging* techniques, as well as by *in vivo* fluorescent microscopy. Furthermore, we demonstrated that the activity and the stability of the enzymes of interest (*Ss*pCA, the *α*-carbonic anhydrase from *Sulfurihydrogenibium yellowstonense*; and *Ss*βGly, the *β*-glycoside hydrolase from *S. solfataricus*) fused to the ASL*^tag^* and exposed on the surface of *E. coli* cells were not affected by the presence of this novel *protein-tag*.

## 2. Materials and methods

### 2.1. Reagents

All DNA restriction and modification enzymes and the fluorescent substrate for the OGT activity (SNAP-Vista Green™, hereinafter BG-FL) were purchased from New England Biolabs (Ipswich, MA); molecular biology kits for the plasmid preparations and DNA gel extractions (NucleoSpin^®^ Gel and PCR Clean-up^®^) were from Macherey-Nagel GmbH (Germany); Lyophilised Thrombin Protease from GE Healthcare (Illinois, US). Eurofins Genomics (Germany) performed the oligonucleotides synthesis and the DNA sequencing service.

### 2.2. DNA constructs

To obtain the pET-ASL*^tag^* construct, we replaced the α-carbonic anhydrase (*Ss*pCA) gene with the *ogt*H^5^ gene in the previously described vector pET-22b/INPN-*Ss*pCA^30^. By the latter and the pQE-*ogt*H^5^ ^35^ plasmid as template, the DNA fragments relative to the INPN domain and H^5^ were respectively amplified with the INPN- and H^5^-Fwd/Rev oligonucleotides pairs (listed in Table 1), under the following conditions: an initial denaturation at 95.0 °C for 5 min, 30 cycles of 30 s at 95.0 °C, 30 s at 50.0 °C and 30 s at 72.0 °C, followed by a final extension of 5 min at 72.0 °C. DNA products were fused to each other in a further PCR amplification, taking advantage of the total complementarity of the INPN-Rev to the H^5^-Fwd oligonucleotide, obtaining the final ASL*^tag^* DNA fragment. Subsequently, this fragment and the pET-22b/INPN-*Ss*pCA vector were digested with Hind III and Xba I restriction endonucleases, gel-purified, and ligated. The ligation mixture was used to transform the *E. coli* DH5α strain, and positive colonies were confirmed by colony PCR and DNA sequencing.

**Table 1.** Oligonucleotides used in this study.

Oligonucleotide	Sequence
INPN-Fwd	5′-TAATACGACTCACTATAGGG-3’
INPN-Rev	5′-GGTGATGGTGAGATCCTCTCGGAACCAGAGATCCATAGGCTTCAATCAGATCGC-3′
H^5^-Fwd	5′-GCGATCTGATTGAAGCCTATGGATCTCTGGTTCCGAGAGGATCTCACCATCACC-3′
H^5^-Rev	5′-TCACCTTCATATGACCATTCATGTTCGCTACCATAAGCTTCTGTCGACGGTACCTCGAGTTCTGG-3′
H^5^-Rev2	5′-ATTGAGCAACTGACTGAAATGCC-3′
*Ss*pCA-Fwd	5′-CCAGAACTCGAGGTACCGTCGACAGAGGCTTATGGTAGCGAACTGAATGGTCATATGAAGGTGA-3′
*Ss*pCA-Rev	5′-CTAGTTATTGCTCAGCGGT-3′

The same cloning strategy was used to achieve the pET-ASL*^tag^*-*Ss*pCA construct: the ASL*^tag^* DNA fragment obtained this time using the H^5^-Rev2 oligonucleotide was used as template to further fuse to the *Ss*pCA gene (obtained by amplification with *Ss*pCA Fwd/Rev oligonucleotide pairs, Table 1). Again, positive colonies after ligation and transformation were confirmed as above.

Finally, the pET-ASL*^tag^*-*lacS* plasmid preparation started with the achievement of the DNA fragment relative to the *ogt*H^5^ gene fused to the β-glycoside hydrolase from the thermophilic archaea *Sulfolobus solfataricus* (*lacS*), obtained by the Hind III/BamH I digestion from the pQE-*ogt*H^5^-*lacS* plasmid^36^. The pET-ASL*^tag^* plasmid was similarly digested to obtain the pET22 recipient for the first ligation/transformation round. Positive blue colonies were selected by the hydrolase activity of the *lacS* gene product (*Ss*βGly) on the Ampicillin selective Luria-Bertani (LB) Agar plates supplemented with 5-bromo-4-chloro-3-indolyl-β-D-glucopyranoside (X-Glc), and the insertion confirmed using PCR analysis. This intermediate vector was further digested with BamH I, treated with Alkaline Phosphatase Calf Intestinal (CIP) and ligated to the BamH I/BamH I INPN DNA fragment, derived from the digestion of the pET-ASL*^tag^* plasmid. In this case, positive blue colonies on LB-Amp-X-Glc agar plates were confirmed by Pst I restriction enzyme digestion analysis.

### 2.3. Determination of the protein expression by a fluorescent assay based on the H^5^ activity

All constructs were used to transform *E. coli* BL21(DE3) cells. Cultures were grown at 37.0 °C in LB selective medium supplemented with 100.0 mg/L ampicillin and 30.0 mg/L chloramphenicol; expression was induced with 1.0 mM isopropyl-thio-β-D-galactoside (IPTG) when an absorbance value of 0.5–0.6 A_600 nm_ was reached, or in the ZY auto-induction medium (AI)^38^, supplemented with the same selective antibiotics. After an overnight incubation, whole cells were collected and assayed by using the BG-FL fluorescent H^5^ substrate previously described^35–37^ ^,^ ^39^ for a qualitative measurement of the protein expression, an aliquot of 1.0 mL of cells was centrifuged at 4000 × *g* and the pellet was resuspended in 50.0 μL of 5.0 μM BG-FL in phosphate-buffered saline (PBS 1×, 20.0 mM phosphate buffer, NaCl 150.0 mM, pH 7.3). After an incubation for 2.0 h at 37.0 °C, reactions were stopped by denaturing the samples in Leammli Buffer 1× and directly loaded on SDS-PAGE, followed by *gel-imaging* on a VersaDoc 4000™ system (Bio-Rad), by applying a blue LED/530 bandpass filter as excitation/emission parameters, respectively. Finally, the fluorescence intensity of each band was normalised to the intensity of the signal obtained from the Coomassie Blue staining analysis.

For a quantitative determination of the expression, whole cells were opportunely diluted to achieve an OD_600nm_ of 1.0. By following the same above-mentioned assay, three different volumes of whole cells were loaded *in the same gel* with defined amounts (in the range of 0.8–50.0 pmols) of purified free H^5^ protein, after the reaction on BG-FL in the same conditions. The obtained values of the relative fluorescence as a function of the purified loaded H^5^ were fitted by a linear equation, whose parameters were then used for the estimation of the amount of expressed H^5^-derivated fusion proteins, assuming that the activity of the H^5^ moiety in the fusions is not affected by the presence of the other protein partner(s). Given that the concentration of *E. coli* cell cultures of 1.0 OD_600nm_ is ca. 8.0 × 10^8^ cells/mL, and the amount of wet cells is 1.7 g/L, it is possible to calculate the yield of expressed proteins in terms of pmol/mg of cells^40^.

### 2.4. Membranes fractionation

The *E. coli* outer (OM) and inner (IM) membranes were purified by following a procedure previously described^27^. Briefly, harvested bacterial cells were resuspended 1:20 (g/mL) in 25.0 mM Tris/HCl buffer, pH 8.0 and disrupted by sonication on ice (10 cycles of 10 s: 50 s on:off treatment). Cell extract was centrifuged at 120,000 × *g* for 1.0 h, and the supernatant containing the cytoplasmic fraction was discarded. Both IM and OM fractions were recovered in the pellet and resuspended in 20.0 mL of PBS 1×, containing 0.01 mM MgCl_2_ and 2.0% Triton X-100. After incubation at room temperature for 30.0 min, the solution was centrifuged as described above. Then, a defined amount of the OM fraction obtained was assayed for the H^5^ activity, leading to the quantitative determination of the total amount of fusion protein, as previously described.

### 2.5. Thrombin assay

The ASL*^tag^* on the OM was cleaved with the Thrombin Protease, in order to separate the H^5^ moiety from the INPN transmembrane domain. A suspension of *E. coli* whole cells was gently centrifuged at 3000 × *g* for 10.0 min at 4.0 °C and then resuspended in a half volume of PBS 1×. The sample was incubated with 30.0 U of Thrombin Protease at 25.0 °C overnight under gentle agitation in the presence of 5.0 μM of BG-FL, followed by the same centrifugation. Cells and supernatants were separately loaded on SDS-PAGE and analyzed by *gel-imaging* and Coomassie staining as described.

### 2.6. Microscopy analysis of *E. coli* cells

For *in vivo* imaging, *E. coli* BL21(DE3) cells transformed with pET-22b/INPN-*Ss*pCA or pET-ASL*^tag^* plasmids were IPTG-inducted, grown overnight at 37.0 °C and finally diluted until OD_600nm_ of 1.0. An amount of cells corresponding to a volume of 1.0 mL of the culture was washed twice in PBS 1× and finally resuspended in 50.0 μL of the same buffer supplemented with 5.0 μM of the BG-FL substrate. After an incubation at 37.0 °C for 30.0 min, cells were washed twice, resuspended and again incubated for 30.0 min at 37.0 °C, to allow the external diffusion of the unreacted substrate. Labelling was first verified by fluorescence *gel-imaging* on SDS-PAGE and then spotted on poly-L-lysine coated slides for microscopy analysis.

Images were collected using a DM6 fluorescence microscope and Hamamatsu camera under the control of Leica LAS AF 6000 software; excitation and emission wavelengths used suitably for AlexaFluor488 dye were *λ*_ex_ = 490 nm; *λ*_em_ = 525 nm, respectively.

### 2.7. ***β***-glycoside hydrolase assay

The β-glycoside hydrolase assay was performed as previously described^41^ at different temperatures in 50 mM sodium phosphate buffer at pH 6.5, in the presence of 2 Np- and 4 Np-Glc substrates at 5.0 mM final concentration. OM fractions containing ASL*^tag^* and relative fusion proteins amounts ranging from 1.0 to 5.0 μg were used in each assay. For the correction of the spontaneous hydrolysis of the substrates, all the reactants except the enzyme (blank mixture), was taken into account. The enzymatic activity was calculated on the basis of the molar extinction coefficient (ε_M_) values of 2- and 4-nitrophenol in 50 mM sodium phosphate buffer pH 6.5 at different temperatures, as previously reported^41^. We defined as one unit of enzyme activity the amount of enzyme hydrolyzing 1.0 μmol of substrate in 1.0 min, under the above-described conditions.

### 2.8. Carbonic anhydrase assay

CA activity assay was a modification of the procedure described by Capasso *et al*.^33^. Briefly, the hydratase assay was performed at 0 °C using CO_2_ as substrate following the pH variation due to the catalyzed conversion of CO_2_ to bicarbonate. Bromothymol blue was used as pH indicator. The production of hydrogen ions during the CO_2_ hydration reaction lowers the pH of the solution leading to a colour transition of the dye. The time required for the colour change is inversely proportional to the amount of CA present in the sample. The Wilbur–Anderson units (WAU) were calculated according to the following definition: one WAU of CA activity is defined as the ratio (*T*_0_ − *T*)/*T*, where *T*_0_ (the time needed for the pH indicator color change for the uncatalyzed reaction) and *T* (the time needed for the pH indicator color change for the catalyzed reaction) are recorded as the time (in seconds) required for the pH to drop from 8.3 to the transition point of the dye (pH 6.8) in a control buffer and the presence of enzyme, respectively.

## 3. Results and discussion

### 3.1. Expression analysis and localisation of ASL^tag^ in *E. coli*

The *in vivo* alkyl-transferase fluorescent assay of H^5^ on whole bacterial cells is a useful method to determine the heterologous expression of this protein and/or relative fusion proteins in the mesophilic *E. coli* and in the thermophilic species *T. thermophilus* and *S. islandicus*, without any purification procedure^35–37^. The *in vivo* assay is possible since the bacterial cells are permeable to OGT fluorescent substrates (as the commercially available SNAP-Vista Green™ or SNAP-Cell TMR™); and the catalytic activity at mesophilic temperatures (25–37 °C) of H^5^ mutant is one order of magnitude higher than the *Ss*OGT wild-type counterpart and comparable to the commercial SNAP-*tag*™^36^. Despite GFPs utilisation, the covalent conjugation of H^5^ with the benzyl-fluorophore moiety of the substrate (Figure 2) allows the denaturation of the whole cells and the direct loading of the samples on SDS-PAGE for the *gel-imaging* analysis, as described in the Materials and Methods^35–37^ ^,^ ^39^.

**Figure 3.**
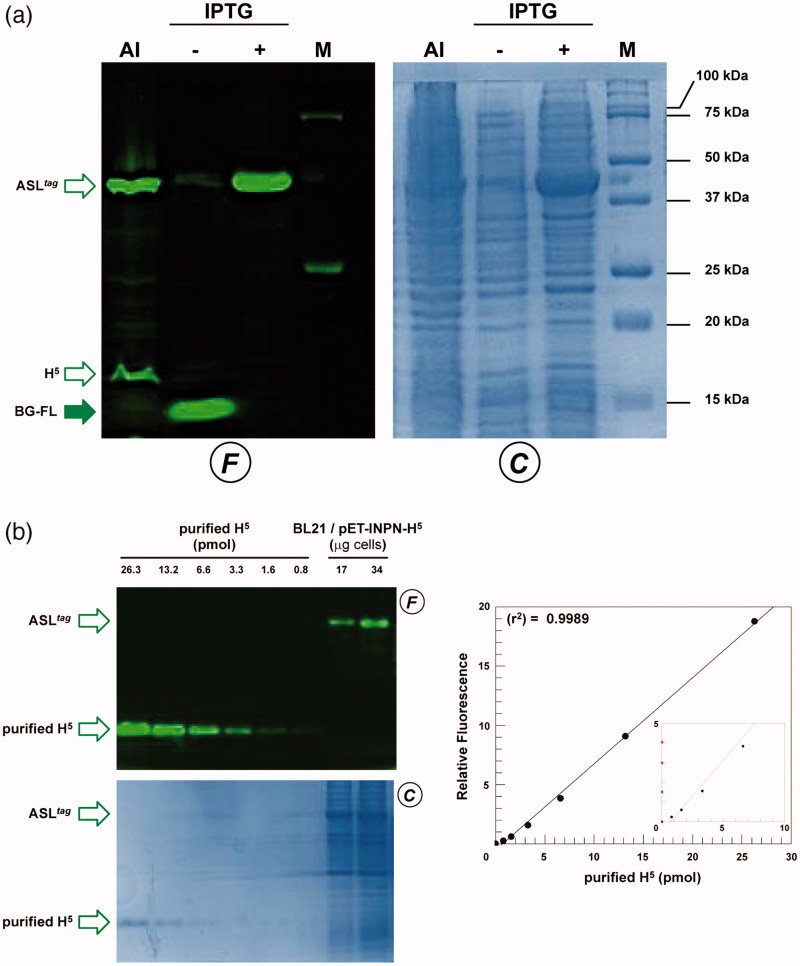
The ASL*^tag^* expression in *E. coli*. (a) *E. coli* BL21(DE3) strain transformed with the pET-ASL*^tag^* plasmid was grown in IPTG-inducted or in auto-induction medium (AI). After the *in vivo* OGT assay, a defined amount in micrograms of whole cells at OD_600nm_ of 1.0^40^ was directly loaded on SDS-PAGE, followed by *gel-imaging* fluorescence (F) and Coomassie staining (C) analyses. Open and closed green arrows indicate fluorescent signals of free H^5^ or H^5^-based fusion proteins, and the free BG-FL substrate, respectively. M: molecular weight marker. (b) Quantitative estimation of the ASL*^tag^* expression: defined amount of cells and purified H^5^ protein (in pmols) were loaded and analyzed on a SDS-PAGE (*on the left*). Fluorescent values obtained from H^5^ were fitted in a linear plot (*on the right*), as described in Materials and Methods. Obtained parameters allowed the quantitative determination of the amount of ASL*^tag^* in *E. coli* cells and shown in Table 2.

**Figure 4.**
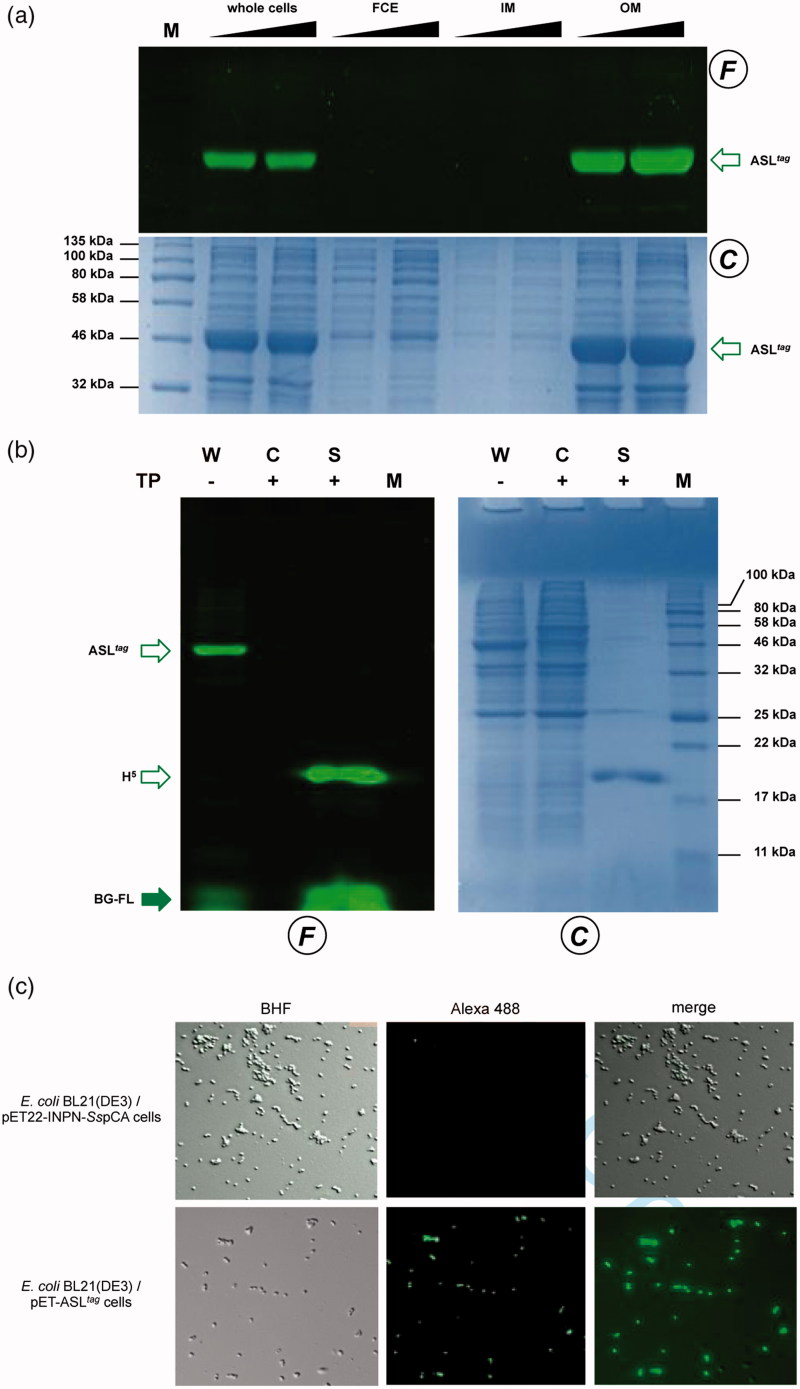
Localisation of ASL*^tag^* in *E. coli*. (a) *gel-imaging* and Coomassie staining analyses after SDS-PAGE of different loaded amount of the whole cells, the relative cytoplasmic fraction (FCE), the inner (IM) and the outer membrane (OM) fractions. (b) Cleavage of ASL*^tag^* by the Thrombin protease (T) on whole cells (W). After the H^5^ reaction during the protease treatment, cells were centrifuged, separating the supernatant (S) from the intact cells (C). (c) *E. coli* BL21(DE3) cells transformed with pET-22b/INPN-*Ss*pCA (Top) or with pET-ASL*^tag^* (bottom) were incubated with BG-FL and then analyzed at fluorescence microscopy. Images show bright field (BHF), AlexaFluor488 (green) and merged images. All used symbols are described in Figure 3(a).

To evaluate the expression of the ASL*^tag^*, *E. coli* BL21(DE3)/pET-ASL*^tag^* cells were grown in LB medium and induced with IPTG or in the AI. In the latter case, although the expression level of the fusion protein was satisfactory, the presence of only the H^5^ signal (ca. 20% of the total fluorescence intensity) in the *gel-imaging* analysis is difficult to rationalise (Figure 3(a)). Probably, during the expression of the protein (e.g., in the advanced stage of the growth) could occur interruptions or failures in the translocation process of the ASL*^tag^* on the outer membrane of *E. coli* with following breaking/cleavage events, especially in the spacer region between INPN and H^5^ (Figure 2). On the other hand, after the IPTG induction, a strong and clear signal at the expected molecular weight is visible, without any fragmentation (Figure 3(a)). This is an important result since, to date, the heterologous expression of *Ss*OGT wild-type and relative variants in *E. coli* has been generally performed in the ABLE C strain, which keeps low the number of copies of the *ogt*-containing plasmids^35^ ^,^ ^36^. As proof, transformed *E. coli* BL21(DE3) strain with the pQE-*ogt*H^5^ plasmid^36^ showed a very low expression of free H^5^ (Supplementary Figure S1), whereas the fusion with the INPN domain and the consequent translocation on the outer cell membrane made the H^5^ expression possible in this strain. The comparative fluorescent analysis with a defined amount of free H^5^ enzyme, allowed us to quantitatively measure the heterologous expression of ASL*^tag^* as pmol/mg of the whole wet cells (Figure 3(b) and Table 2). The assay on H^5^ confirmed the *anchoring* function of the INPN trans-membrane protein: only the whole cells and the fraction containing the outer membrane displayed a fluorescent band corresponding to the ASL*^tag^* fusion protein, whereas the signal was missed in the lanes relative to the cytoplasmic and the inner membrane fractions (Figure 4(a)). Besides, the evidence of the INPN on the bacterial outer membrane was confirmed by treating the whole bacterial cells with the thrombin, too (Figure 2). A cleavage site for this protease was localised between the INPN domain and the H^5^ moiety (Figure 2). The fluorescent signal corresponding to the MW of the H^5^ protein was present only in the supernatant fraction when the protease was added at the same time with the BG-FL substrate (Figure 4(b)). Finally, we checked the OGT activity of ASL*^tag^* in living cells by microscope analysis, upon the labelling with the fluorescent substrate. The obtained images of living *E. coli* BL21(DE3) cells showed a strong and specific fluorescent signal only in those transformed with the ASL*^tag^*-containing plasmid (Figure 4(c)), indicating that the fusion protein is stable and proficient to labelling. This data suggest that ASL*^tag^* is suitable for localisation and analysis of membrane proteins, and provide an opportunity for further *in vivo* analyses of ASL*^tag^*-tagged proteins of interest under physiological conditions.

**Table 2.** Quantitative estimation of the heterologous expression of ASL*^tag^* and relative fusion proteins in *E. coli* BL21(DE3) whole wet cells.

	MW^a^ (kDa)	ASL*^tag^-*E**ratio	Fusion yield^b^ (pmol H^5^/mg)	E yield^c^ (μg/mg)	(*r*^2^)^d^
ASL*^tag^*	42.0	–	152.5 ± 15.7	–	0.9977
ASL*^tag^*-*Ss*pCA	70.2	1:1	58.4 ± 11.6	1.54 ± 0.30	0.9986
ASL*^tag^*-*Ss*βGly	98.8	4:1	30.6 ± 13.2	7.2 ± 3.1^e^	0.9980

a Calculated from the primary structure.

b On the basis of the H^5^ activity on fluorescent *gel-imaging* analysis (see Materials and Methods).

c The amount of the immobilised enzyme without the ASL*^tag^*.

d Correlation coefficient of the linear curve obtained from the H^5^ values of fluorescence as a function of the amount of the loaded protein.

e The tetrameric form of the catalytically active *Ss*βGly enzyme.

### 3.2. *In vivo* immobilisation of thermostable enzymes by ASL^tag^

**Figure 5.**
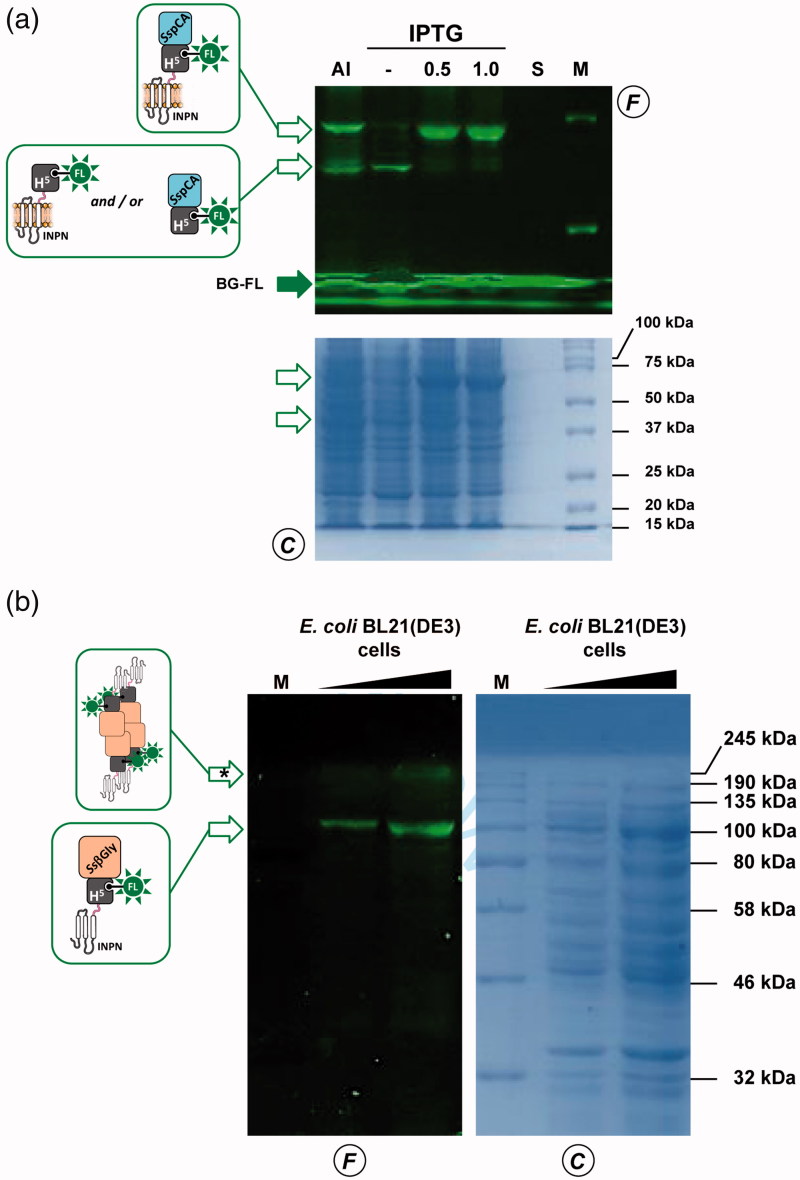
Heterologous expression of thermostable enzymes fused to ASL*^tag^.* SDS-PAGE analysis of the ASL*^tag^*-*Ss*pCA (a) and ASL*^tag^*-*Ss*βGly (b) expression. (a) Fluorescent signals on the F panel correspond to bands whose molecular weights are attributable to the protein fusions shown in the schemes. (b) 1.0 mM IPTG-inducted cells gave a very high fluorescent band (marked by an asterisk), presumably corresponding to the tetrameric form of *Ss*βGly linked to 4 × ASL*^tag^* units, as shown. All used symbols are described in Figure 3(a).

As described in the literature, it has been demonstrated that the monomeric α-carbonic anhydrase (*Ss*pCA) from the thermophilic bacterium *S. yellowstonense* can be actively expressed onto the outer membrane of *E. coli* ^30^. Following this strategy, we realised a plasmid expressing the ASL*^tag^*-*Ss*pCA construct by inserting the *ogt*H^5^ gene between the INPN and *Ss*pCA. The expression profile analyzed by following the H^5^ activity on BG-FL confirmed that in the AI multiple fluorescent signals are present (Figure 5(a)), mainly represented by the full-length ASL*^tag^*-*Ss*pCA (70.2 kDa; ca. 65% of the total fluorescence intensity) and lower band (ca. 35%) closer to 37 kDa than 50 kDa (Figure 5(a)). This signal is compatible to the ASL*^tag^* (42.0 kDa) as well as the H^5^-*Ss*pCA moiety (46.0 kDa), suggesting that both translation interruption and translocation failure events in this growth conditions can be not excluded. Again, we detected an optimal achievement of the full-length fusion protein under IPTG-based expression in LB medium (ca. 95%; Figure 5(a)). In this condition and considering the INPN:H^5^:*Ss*pCA ratio as 1:1:1, the amount of the whole fusion protein expressed was estimated as ca. 60.0 pmol/g cells, which corresponds to ca. 1.5 μg of the sole immobilised carbonic anhydrase per mg of cells (Table 2). Preliminary assays on ASL*^tag^*-*Ss*pCA indicated that the presence of H^5^ does not hamper the hydratase activity of the *Ss*pCA, if compared with that of the previously expressed INPN-anchored enzyme^30^ (data not shown).

ASL*^tag^* system was also tested with another thermostable enzyme, the β-glycoside hydrolase (*Ss*βGly) from the thermophilic archaea *S. solfataricus* ^42^. We previously demonstrated that the cytoplasmic H^5^-*Ss*βGly fusion protein is stable and active for both the OGT and the β-glycosidase assays, suggesting that the presence of one enzyme does not interfere with the folding and activity of the other^36^. Interesting to note that anchoring this protein fusion to the bacterial outer membrane by the INPN domain is particularly challenging because *Ss*βGly is active only in its tetrameric form^43^ ^,^ ^44^. The presence of blue colonies on LB agar plate in the presence of a glucoside chromogenic derivative (X-Glc), which is a preferred substrate of *Ss*βGly^41^ but not of the *E. coli* LacZ (a β-galactosidase enzyme) was a first and convincing indication of the oligomerisation of this thermostable enzyme. Although in this case the amount of the expressed fusion protein was lower than the above examples (Figure 5(b) and Table 2), IPTG-inducted *E. coli* BL21(DE3)/pET-ASL*^tag^*-*Ss*βGly cells displayed a fluorescent signal of expected molecular weight (98.8 kDa), corresponding to one monomer of *Ss*βGly fused to one ASL*^tag^* unit. However, a higher band is clearly visible in the fluorescence analysis (marked with an asterisk in Figure 5(b)), out of the molecular weight marker range used: as for the cytoplasmic H^5^-*Ss*βGly fusion, it could suggest that it corresponds to a partially denatured part of the tetrameric form of ASL*^tag^*-*Ss*βGly (ca. 400.0 kDa), which is particularly resistant to thermal denaturation^36^ ^,^ ^42^ ^,^ ^43^ ^,^ ^45^. Finally, an amount of 0.24 μg/mg of immobilised *Ss*βGly on the OM fraction (on the basis of the calculated H^5^ pmol) was assayed on 2 Np- and 4 Np-Glc at three different temperatures. The results show an activity of 12.6 ± 0.7 and 8.8 ± 0.4 (50 °C), 30.3 ± 0.4 and 20.3 ± 0.9 (60 °C), 51.5 ± 0.9 and 30.8 ± 1.5 U/mg (70 °C), respectively, whereas OM fraction containing the sole ASL*^tag^* did not result in any β-glucosidase activity, as expected (data not shown). These values are correctly related to the activity of *Ss*βGly^45^, clearly indicating that the formation of the quaternary structure on the *E. coli* OM occurs. However, since the 3D structure of this thermostable glycoside hydrolase showed that it is not laying on a surface^43^, we hypothesised an invagination of the *E. coli* outer membrane to allow the assembly of four units of the ASL*^tag^*-*Ss*βGly (Supplementary Figure S2).

## 4. Conclusions and perspectives

**Figure 6.**
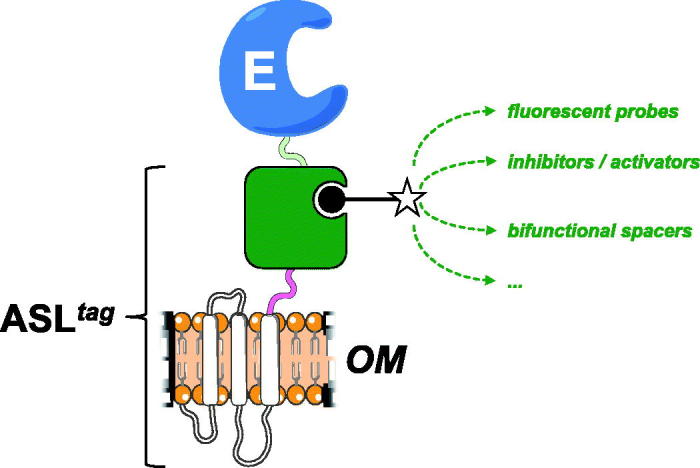
The biotechnological potential of the ASL*^tag^.* Any chemical group of interest (*open star*) conjugated to the BG-substrate (*black closed circle*) could be covalently bound to the H^5^ moiety (*in green*) of the ASL*^tag^*. This enhances the potential use of an immobilised enzyme (E) on the *E. coli* surface (OM), making available to it a series of molecules, e.g., fluorescent probes and enzymatic activity modulators, or bi-functional groups for cascade reactions with other biocatalysts.

In the present work, we introduced and demonstrated the utility of a novel *protein-tag*, composed by the N-terminal domain of the INP protein fused to a DNA repair enzyme. From our results, it is readily apparent that ASL*^tag^* offers: (i) an easy expression and *in vivo one-step procedure* of enzyme immobilisation on biological supports (e.g., *E. coli* outer membrane); (ii) significantly reduces the costs of the enzyme purification and those of the immobilisation support, allowing a direct exposition of the enzyme to the solvent^20^ ^,^ ^30^; (iii) an indirect labeling, by the reaction of a thermostable variant of the SNAP-*tag*™ (H^5^)^46^ ^,^ ^47^, which covalently links desired chemical groups conjugated to its benzyl-guanine substrate^14^ ^,^ ^35^. ASL*^tag^* favoured the expression of a monomeric protein (e.g., the thermostable *Ss*pCA) and an enzyme having a complex quaternary structure (e.g., the thermophilic *Ss*βGly), without compromising their overall folding and enzymatic activity. Moreover, we showed that the utilisation of a fluorescein-derivative of the BG led to the localisation and the quantitative determination of the yield of the expressed ASL*^tag^* and the relative fusion proteins (Table 2). On the other hand, despite the GFPs utilisation limited only to all fluorescence-based applications, the possibility to conjugate different groups to the BG- for the H^5^ reaction^36^ dramatically expands the biotechnological potential of this novel *protein-tag*. For example, it will be possible to modulate the activity of biocatalysts (by introducing inhibitors/activators), or connecting them with other proteins for the improvement of cascade reactions in the presence of bi-functional chemical groups (Figure 6).

## Supplementary Material

Supplemental Material
