# Supplementary material for: An AGT-based protein-tag system for the labelling and surface immobilization of enzymes on E. coli outer membrane
Source: J Enzyme Inhib Med Chem. 2019 Feb 6;34(1):490–9. doi: 10.1080/14756366.2018.1559161 (PMC6366409; doi:10.1080/14756366.2018.1559161)
Supplement: Supplemental Material [file IENZ_A_1559161_SM6112.pdf]

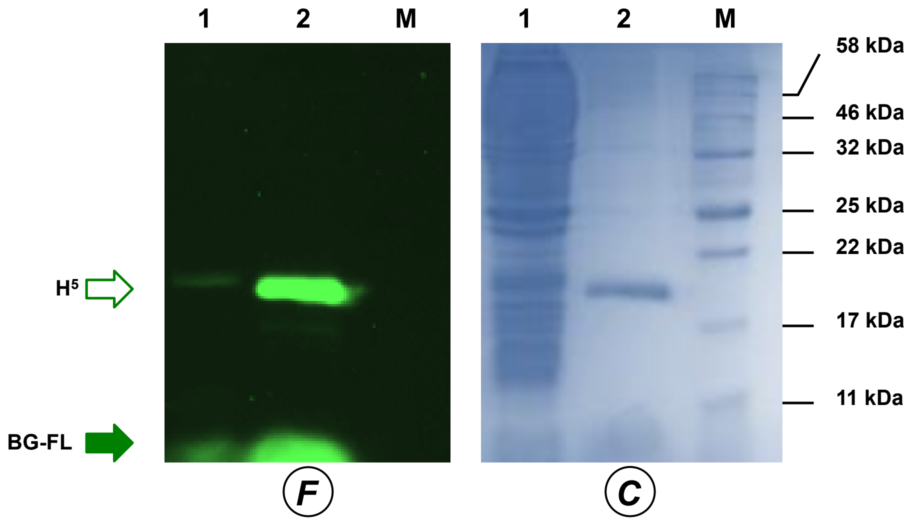

Figure S1. *Expression of free H^5 in BL21(DE3) strain.* Whole IPTG-inducted cells transformed with pQE-*ogt*H^5 plasmid (lane 1)[36] were incubated and loaded on SDS-PAGE for the *gel-imaging* and Coomassie staining analyses, as described in the Materials and Methods. Lane 2 corresponds to the 1.0 μg of the purified free H^5 enzyme. M, molecular weight marker. All used symbols are described in Figure 3a.

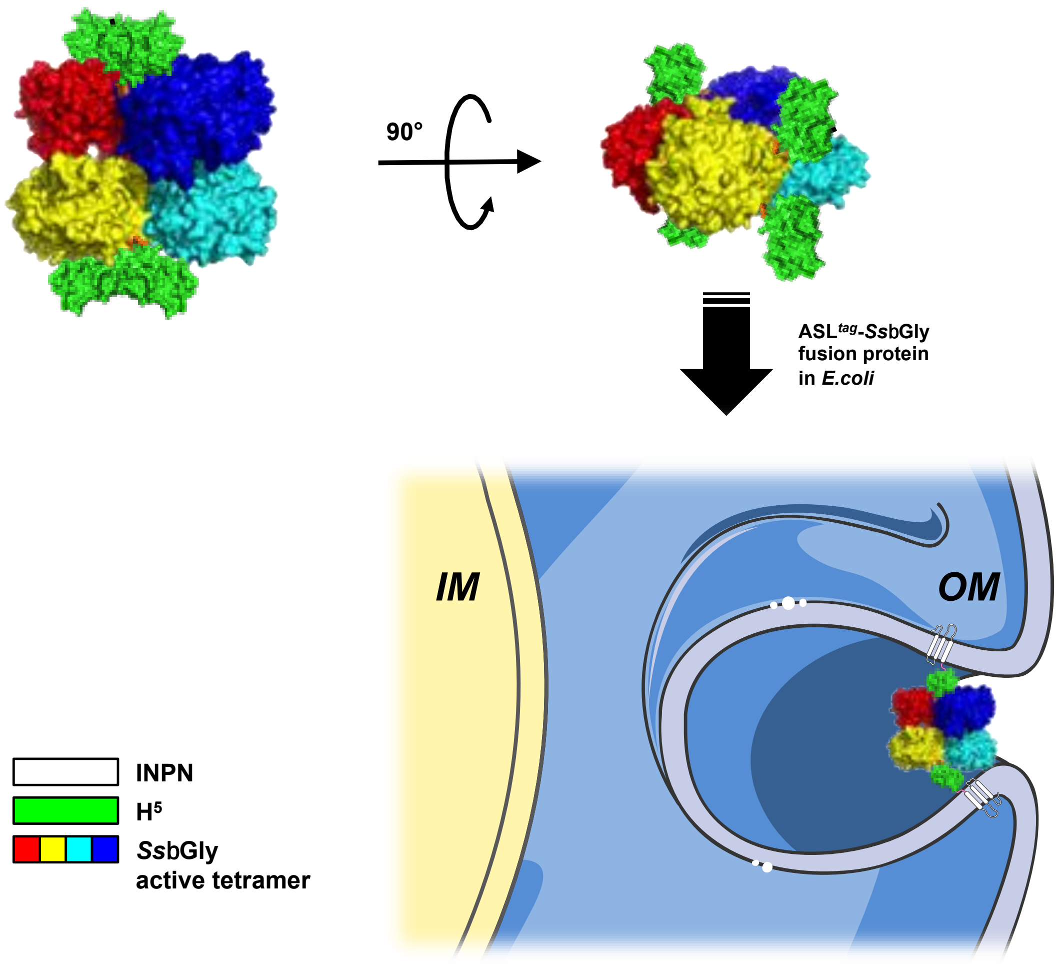

Figure S2. *The tetrameric form of the ASL^{tag}-SsβGly fusion protein.* Schematic representation of the possible spatial disposition of the tetrameric *Ss*βGly (PDB ID: 4GOW[43]) linked to four H^5 units (PDB ID: 6GA0[47]), taking into account of the exposed first methionine residue of each *Ss*βGly monomer (*in orange*). The hypothesized invagination of the external membrane of *E. coli* would makes it possible the assembling of the tetrameric form and the consequent measured catalytic activity of the *Ss*βGly.
